# Revisiting the species list of freshwater fish in Israel based on DNA barcoding

**DOI:** 10.1002/ece3.10812

**Published:** 2023-12-20

**Authors:** Roni Tadmor‐Levi, Tamar Feldstein‐Farkash, Dana Milstein, Daniel Golani, Noam Leader, Menachem Goren, Lior David

**Affiliations:** ^1^ Department of Animal Sciences Robert H Smith Faculty of Agriculture, Food and Environment, The Hebrew University of Jerusalem Rehovot Israel; ^2^ National Natural History Collections, Department of Ecology, Evolution and Behavior The Hebrew University of Jerusalem Jerusalem Israel; ^3^ The Steinhardt Museum of Natural History and School of Zoology Tel Aviv University Tel Aviv Israel; ^4^ Science and Conservation Division Israel Nature and Parks Authority Jerusalem Israel

**Keywords:** COI, fish biodiversity, Middle East, species conservation, species identification, taxonomy

## Abstract

Israel's region forms a continental bridge; hence, the freshwater fish fauna in Israel consists of unique populations of species that originated from Africa, Asia, or Europe and are often endemic or at the edge of their distribution range. Worldwide, fish biodiversity suffers significantly from pressures and disturbances of freshwater habitats, especially in arid regions, such as in parts of Israel. Biodiversity conservation requires efficient tools for monitoring changes in populations. DNA barcoding, by complementing and enhancing species identification, provides such monitoring tools. In this study, over 200 specimens representing over 28 species were DNA barcoded and together with previously available records, a DNA barcoding database for freshwater fish of Israel was established. Of the 71 distinct barcodes generated, 37% were new, attesting to the uniqueness of fish populations in Israel. For most species, morphological and molecular species identifications agreed. However, discrepancies were found for five genera. Based on DNA barcoding, we propose *Acanthobrama telavivensis* as a junior synonym for *Acanthobrama lissneri*. In *Garra* spp., we propose splitting *Garra nana* into two species and assigning *Garra rufa* in the region to *Garra jordanica*, or possibly to two species. Israeli *Pseudophoxinus kervillei* is not the same species as in Syria and Lebanon. However, *Pseudophoxinus syriacus* might not be endangered since it is genetically very similar to *Pseudophoxinus drusensis*. In Israel, instead of five reported *Oxynoemacheilus* species, combining DNA barcoding with morphology suggests only three. Genetic and geographic separation suggested that *Aphanius mento* is likely a species complex. The study provides a thorough barcoding database, suggests significant species reconsiderations in the region, and highlights the Sea of Galilee and the Beit She'an valley streams as biodiversity “hotspots.” This study will therefore promote further studying of the fish species in the region and their ecology, as well as the monitoring and conservation of freshwater fish biodiversity in Israel and the region.

## Graphical abstract

This study established a DNA barcoding database for freshwater fishes in Israel. Molecular and morphological species identifications were compared, and discrepancies were found for samples in five genera, which suggests significant reconsiderations to the species list in Israel and the region. The results highlight the Sea of Galilee and the Beit She'an valley streams as biodiversity “hotspots,” promoting further studying of the fish species in the region and their ecology, as well as monitoring and conservation of freshwater fish biodiversity in Israel and the region.

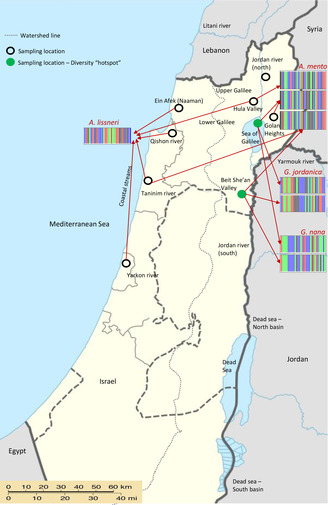

## 1 INTRODUCTION

Freshwater bodies are an essential resource supporting not only aquatic organisms but also terrestrial life. Various human uses of freshwater, such as for drinking, irrigation, hydro‐electric power production and fishing, as well as human derived water pollution, have placed significant pressure on freshwater habitats and freshwater fish communities (Darwall & Freyhof, 2015; Dudgeon et al., 2006; Dynesius & Nilsson, 1994; Lind et al., 2012; Nilsson et al., 2005). Despite the ongoing global decline in freshwater fish diversity, measures to mitigate human‐derived pressures and conserve fish species and habitats have been too few and too slow (Darwall & Freyhof, 2015; Dudgeon et al., 2006; Lind et al., 2012; Sala et al., 2000). Key to effective management of species biodiversity are tools for efficient assessment and monitoring of changes in fish populations (Dudgeon et al., 2006; Levêque et al., 2007; Reid et al., 2019; Sala et al., 2000).

Israel is part of a continental bridge connecting Africa, Asia, and Europe. As such, despite being a small country with a semiarid to arid climates, Israel has a rich fish fauna originating from all three continents. This fauna includes some endemic species, but mainly species which are distributed also in neighboring countries, yet their population in Israel is unique as they live on the edge of their distribution ranges (Por, 1975). Around 30 native and over 40 non‐native fish species were recorded in inland waters in Israel (Ben‐Tuvia, 1981; Golani et al., 2019, 2023; Golani & Mires, 2000; Golani & Snovsky, 2013; Goren, 1974; Goren & Galil, 2005; Goren & Ortal, 1999; Roll et al., 2007; Snovsky & Golani, 2012). However, these numbers change over time due to the description of new species, introduction of non‐native species and species extinction. *Tristramella sacra* (Günther, 1865) is one species that was endemic to the Sea of Galilee in Israel and was recently considered as extinct (Goren, 2014). In 2019, the list of protected species in Israel was updated to include 13 freshwater fish species out of the ~30 known native species. The Israel Nature & Parks Authority has been working to better understand the ecology and manage the protection of these and other species. A comprehensive taxonomic evaluation was acknowledged as an essential first step.

Taxonomy of fish in Israel and the region has been mainly based on morphological characters and geographic distribution patterns. In recent years, DNA barcoding is being increasingly used for taxonomic and ecological research, as well as for other applications (Beng & Corlett, 2020; Galimberti et al., 2013; Hajibabaei et al., 2007; Hebert et al., 2003; Ko et al., 2013; Kress et al., 2015; Pont et al., 2018; Ratnasingham & Hebert, 2007; Ushio et al., 2018; Valentini et al., 2009; Ward et al., 2005, 2009). For fish, it was established that the sequence of the mitochondrial DNA coded gene, cytochrome *c* oxidase subunit 1 (COI), evolves at a suitable rate for species identification, allowing DNA barcoding to complement and enhance species identification (Bhattacharya et al., 2016; Hebert & Gregory, 2005; Ward, 2009). Species identification by DNA barcoding is useful also for efficiently monitoring freshwater fish communities, since DNA can be obtained directly not only from specimens but also from environmental samples such as water and excrements, known as metabarcoding (Pont et al., 2018; Ushio et al., 2018). The application of DNA barcoding for reliable molecular identification of species requires a reference database that connects DNA sequences to specific species (Ekrem et al., 2007; Weigand et al., 2019). The Barcode of Life Data systems (BOLD, https://www.boldsystems.org) is a comprehensive DNA barcode reference library (Ratnasingham & Hebert, 2007), created to support the use of DNA barcode data and molecular species identification for studying various aspects of biodiversity. A few studies on freshwater fish of Israel have already applied DNA barcoding (Borovski et al., 2018; Geiger et al., 2014; Shirak et al., 2009; Tadmor‐Levi, Borovski, et al., 2022; Tadmor‐Levi, Cummings, et al., 2022), but enhancing the utilization of the method required establishing a more comprehensive DNA barcode reference library for the freshwater fish of Israel.

In this study, we first completed a DNA barcoding database for nearly all the freshwater species of Israel. We then further analyzed the genetic information in combination with morphological identifications and geographic data to reassess the checklist of freshwater fishes of Israel and suggest reconsiderations for several taxonomic groups in the region.

## 2 METHODS AND MATERIALS

### 2.1 Ethics statement

Fish collection was done by electrofishing with a handheld net‐electrode (Electro‐shocker EFKO model FEG 6000 producing up to 350 Volts and 17 Amperes, or similar shocker) or by seine net. Both are approved means of fish surveying in Israel and are covered under permits # 2015/40796 to Dr. Eldad Elron and # 2017/41719 to Dr. Yaron Krotman for sampling of protected natural resources as reviewed and approved by the Israel Nature and National Parks Authority.

### 2.2 Fish sampling

Samples for DNA barcoding were obtained from 54 museum specimens (Steinhardt Museum of Natural History, Tel Aviv University) sampled between 2010 and 2014 complemented by specimens sampled anew from freshwater habitats. For each sampling instance, captured fish were euthanized with a 0.6 mL/L of 2‐phenoxy‐ethanol, fish were kept in immersion for at least 10 more minutes after cessation of operculum movement, as described previously (Leary et al., 2013) and placed in jars with 70% technical Ethanol labeled by date, place coordinates and sampler name. Fish were transferred to the Steinhardt Museum of Natural History, Tel Aviv University, for further recording and identification. Identification to the species level was done using published keys and databases (Fricke et al., 2022; Goren, 1983). All specimens were preserved in 70% technical ethanol and stored at the fish collection of the Steinhardt Museum of Natural History, Tel Aviv University. The list of samples used for DNA barcoding is given in Table S1.

### 2.3 Sampling locations

**FIGURE 1:**
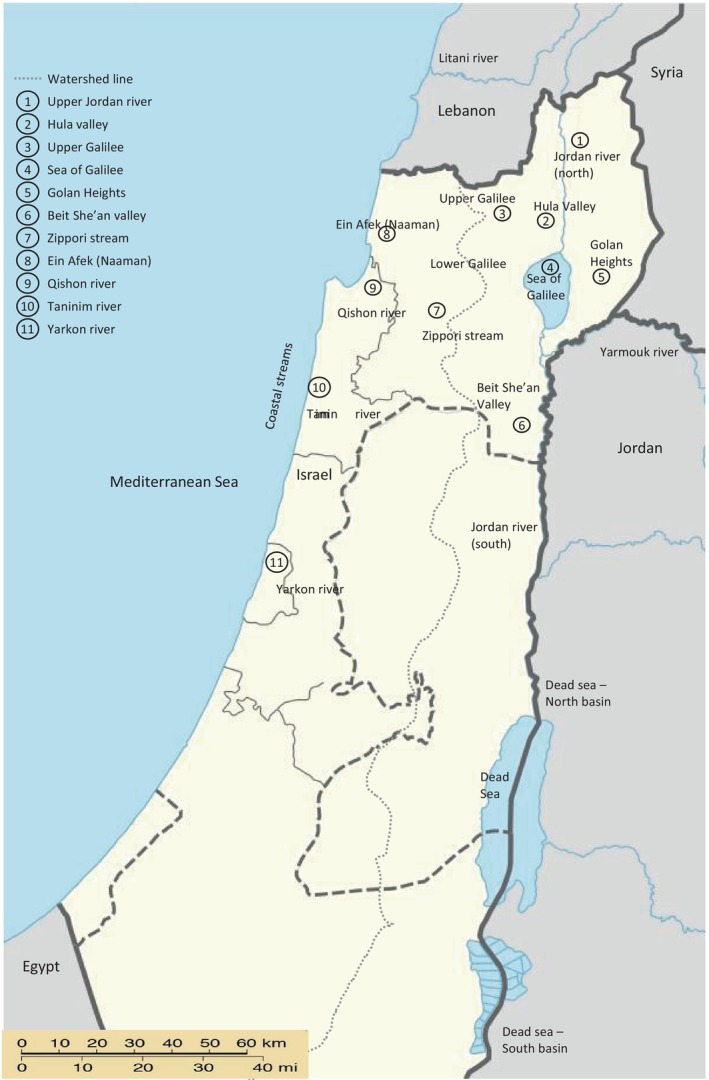
Map of the study region. The map depicts countries from which published DNA barcodes were analyzed and the regions in Israel from which fish were sampled. Note the north‐to‐south watershed line dividing the coastal and the eastern main watersheds of Israel. Map was reprinted from DMY + NordNordWest (https://commons.wikimedia.org/wiki/File:Israel_by_israeli_law_adm_location_map.svg) under a CC BY‐SA 4.0 license, original copyright 2017.

Fish sampling scheme was designed to complement available museum samples and samples from a previous study on the Sea of Galilee fish (Tadmor‐Levi, Borovski, et al., 2022). Freshwater habitats in Israel are divided into western and eastern watersheds, which are separated by a higher altitude division line running north to south (Figure 1). Western watershed rivers and streams flow westwards into the Mediterranean Sea and are called coastal rivers/streams. Eastern watershed rivers and streams flow into the Jordan River basin, which stretches north to south constituting the northern part of the Syrian‐African Rift. Sampling points in the western and eastern watersheds were grouped into regions by their geographic distribution (Figure 1). The western watershed was represented in this study by (from north to south): Ein Afek (Na'aman river), Qishon river and Zippori stream (Lower Galilee), and Taninim river. The eastern watershed was represented by samples from several regions: Jordan River (north) and Hula valley are both part of the northern Jordan River basin, the Sea of Galilee including mouth of Meshushim and Daliyot streams on the east side of this lake, streams of the Golan Heights and the streams of Beit She'an valley as part of the southern Jordan River basin. Details on sampling point for each barcoded sample are given in Table S1 and was deposited on the BOLD data systems under project name FWISR.

### 2.4 DNA isolation

DNA was isolated from a small piece of muscle tissue that was excised close to the anal fin. DNA was isolated using the DNeasy Blood and Tissue Kit (Qiagen) following the manufacturer's protocol with overnight digestion of the tissue.

### 2.5 PCR amplification and sanger sequencing of the barcoding sequence

A fragment of approximately 700 bp from the mitochondrial gene coding for COI was amplified by PCR using a mixture of three degenerate forward primers and one reverse primer slightly modified from (Ward et al., 2005): Fish_F1m 3′‐tcaaccaaycacaaagacattggcac‐5′; Fish_F2m 3′‐tcgactaatcacaaagayatyggcac‐5′; Fish_F3m 3′‐tcaactaatcacaaagayatcggcac‐5′; Fish_R2m (reverse primer) 3′‐acttcagggtgnccraagaatcaraa‐5′. PCR amplification was performed using Ex‐Taq DNA polymerase (Takara Bio.). The PCR profile included denaturation at 94°C for 3 min, followed by 35 cycles of denaturation at 94°C for 30 s, annealing at 52°C for 30 s, and elongation at 72°C for 60 s. The cycles were followed by a final elongation step at 72°C for 10 min. PCR products were cleaned from residual primers with the enzymes Exonuclease I (Exo I) and Alkaline Phosphatase (Thermo Fisher Scientific) and directly sequenced using Big Dye Terminator v1.1 (Applied Biosystems) on an ABI 310 sequencer by the DNA Sequencing Unit at the G.S. Wise Faculty of Life Sciences, Tel Aviv University.

### 2.6 Data analysis

COI sequence files were assembled and aligned using ClustalW algorithm with the BioEdit software (Version 7.2.5; Hall et al., 2011). All the polymorphisms detected by the software were manually curated by examining the chromatograms and dubious variants were manually removed. Successful sequences and sampling details for each sample were deposited on BOLD (https://www.boldsystems.org, Ratnasingham & Hebert, 2007) under project name FWISR. Aligned sequences of each species were manually inspected, and polymorphic sequences within species were defined as different barcode haplotypes. Each haplotype was queried against species level barcode records using BOLD identification module to derive molecular species identification. Additionally, all specimens were clustered into operational taxonomic units (OTUs) based on their COI sequences, using the Refined Single Linkage algorithm (RESL) using BOLD “cluster sequences” tool and Barcode Index Numbers (BIN) system (Ratnasingham & Hebert, 2013). Since we observed full correspondence between independently assigned OTUs and the BIN system accessions, BIN accessions are given to describe the suggested OTUs. For species where morphological and barcoding identification matched, only the DNA barcodes of fish sampled in this study were considered.

However, for species with disagreements between morphological and barcoding identification, barcodes of the same and related species were downloaded from BOLD, aligned with barcodes of this study and further analyzed. Sequence alignments of each disagreement group (genus or species) are given in Files [Link], [Link], [Link], [Link], [Link]. On each of these datasets, a focused species delimitation procedure was performed (as described above for the complete dataset) and an estimated unrooted COI gene tree was constructed utilizing the Molecular Evolutionary Genetics Analysis (MEGA6) freeware (Tamura et al., 2013), using the Kimura‐2‐parameter model (K2P), and the neighbor‐joining (NJ) tree construction method with 1000 bootstrap replicates (Felsenstein, 1985). We further considered genetic distances to nearest neighbor (obtained by the RESL analyses) and in specific cases, calculated minimum, maximum and mean pairwise K2P genetic distance between any pair of samples or groups of samples using MEGA6 software.

## 3 RESULTS

### 3.1 Reference COI database for freshwater fish of Israel

The first aim of this study was to create a reference database of COI DNA barcodes for the freshwater fish of Israel. Successful barcodes were obtained from a total of 205 samples, representing 32 taxonomic groups. Of them, 28 were morphologically identified to the species level and four additional non‐native taxonomic groups were identified to the genus or family levels. Out of the 28 taxonomic groups identified to the species level, 21 were native to the freshwater systems of Israel, while the remaining seven were not (Table 1). The published list of native freshwater fish fauna of Israel also includes five additional species which are currently considered extinct in Israel, and four additional species which were not included in this study (Goren & Ortal, 1999). For three of the latter, *Mirogrex terraesanctae* (Steinitz, 1952), *Luciobarbus longiceps* (Valenciennes, 1842) and *Tristramella simonis* (Günther, 1864), DNA barcode data has been previously reported (Tadmor‐Levi, Borovski, et al., 2022). For the fourth, *Aphaniops richardsoni* (Boulenger, 1907), the sampling scheme did not cover its habitat. A list of the native recorded freshwater species of Israel modified from (Goren & Ortal, 1999) with their current status is given in Table A1 in Appendix 1.

**TABLE 1:** List of barcoded and morphologically identified species, their COI haplotypes and frequencies, their DNA barcoding species identification and their associated BIN cluster in BOLD (* denotes haplotypes new to BOLD and † denotes a non‐native species).

Family	Species (morphology)	*N* samples	COI haplotype	Haplotype frequency	Species (barcoding)	BIN clustering (by BOLD)
Anguillidae	*Anguilla anguilla*	4	An.an_A	0.25	Matches morphological	ADC8854
			An.an_B	0.25	"	"
			An.an_C	0.25	"	"
			An.an_D	0.25	"	"
Blenniidae	*Salaria fluviatilis*	1	Sa.fl_A	1	Matches morphological	ACL6766
Cichlidae	*Amatitlania nigrofasciata* ^†^	2	E_An_A	1	Matches morphological	AAD6407
	*Astatotilapia flaviijosephi*	7	As.fl_A	1	Matches morphological	AAD8533
	Cichlidae sp.^†^	7	Cich_A	0.86	Multiple lake Malawi Cichlids	AAX2832
			Cich_B *	0.14	"	"
	*Coptodon zillii*	11	Co.zi_A *	0.09	Matches morphological	AAB9042
			Co.zi_B	0.18	"	"
			Co.zi_C	0.73	"	"
	*Oreochromis aureus*	7	Or.au_A *	0.14	Matches morphological	AAA6537
			Or.au_B	0.86	"	"
	*Sarotherodon galilaeus*	3	Sa.ga_A	1	Matches morphological	AAA6537
Clariidae	*Clarias gariepinus*	11	Cl.ga_A	0.91	Matches morphological	AEC0350
			Cl.ga_B	0.09	"	"
Cyprinidae	*Acanthobrama lissneri*	8	Ac.li_A *	0.25	*Acanthobrama lissneri/telavivensis*	ACL7484
			Ac.li_B	0.25	*"*	"
			Ac.li_C	0.13	*"*	"
			Ac.li_D *	0.25	*"*	"
			Ac.li_E *	0.13	*"*	"
	*Acanthobrama telavivensis*	3	Ac.te_A	1	*Acanthobrama lissneri/telavivensis*	ACL7484
	*Capoeta damascina*	9	Ca.da_A	0.67	Matches morphological	AAP1452
			Ca.da_B *	0.22	"	"
			Ca.da_C	0.11	"	"
	*Carasobarbus canis*	11	Ca.ca_A	1	Matches morphological	ACL7718
	*Carassius* sp.^†^	1	Ca.sp._A	1	*Carassius auratus*	AAA7176
	*Cyprinus carpio* ^†^	5	Cy.ca_A	0.6	Matches morphological	AAA7175
			Cy.ca_B	0.2	"	"
			Cy.ca_C	0.2	"	"
	*Garra rufa*	9	Ga.ru_A	0.22	*Garra jordanica/rufa*	AAZ7685
			Ga.ru_B *	0.22	*"*	"
			Ga.ru_C	0.44	*"*	ACL7806
			Ga.ru_D *	0.11	*"*	"
	*Garra nana*	11	Ga.na_A *	0.09	Matches morphological	ACL7068
			Ga.na_B *	0.09	"	"
			Ga.na_C *	0.09	"	ACL7216
			Ga.na_D	0.73	"	"
	*Pseudophoxinus drusensis*	4	Ps.dr_A	1	*Pseudophoxinus drusensis/syriacus*	ACL6832
	*Pseudophoxinus kervillei*	8	Ps.ke_A *	0.25	Matches morphological	ADM4499
			Ps.ke_B *	0.13	"	"
			Ps.ke_C *	0.13	"	"
			Ps.ke_D *	0.38	"	"
			Ps.ke_E *	0.13	"	"
Cyprinodontidae	*Aphanius mento*	10	Ap.me_A	0.1	Matches morphological	ACL6470
			Ap.me_B *	0.1	"	AEH8149
			Ap.me_C *	0.1	"	"
			Ap.me_D *	0.2	"	"
			Ap.me_E	0.3	"	ACL6472
			Ap.me_F *	0.1	"	ACL6470
			Ap.me_G *	0.1	"	"
Moronidae	*Morone chrysops* ^†^	1	Mo.ch_A	1	Matches morphological	AAE0607
Mugilidae	*Chelon ramada*	2	Ch.ra_A	1	Matches morphological	AAD5443
	*Mugil cephalus*	5	Mu.ce_A	0.4	Matches morphological	ACE3807
			Mu.ce_B *	0.2	"	"
			Mu.ce_C *	0.2	"	"
			Mu.ce_D	0.2	"	"
Nemacheilidae	*Oxynoemacheilus dori*	9	Oxy_A *	1	*Oxynoemacheilus insignis*	ACL7539
	*Oxynoemacheilus insignis*	11	Oxy_B	0.82	*Oxynoemacheilus leontinae*	ADM1019
			Oxy_C	0.09	Matches morphological	ACL7539
			Oxy_D	0.09	*Oxynoemacheilus leontinae*	ADM1019
	*Oxynoemacheilus jordanicus*	7	Oxy_B	0.86	*Oxynoemacheilus leontinae*	ADM1019
			Oxy_D	0.14	"	"
	*Oxynoemacheilus leontinae*	7	Oxy_E	0.86	Matches morphological	ADM1019
			Oxy_F	0.14	"	"
	*Oxynoemacheilus panthera*	10	Oxy_G *	0.4	*Oxynoemacheilus insignis*	ACL7539
			Oxy_H	0.1	*"*	"
			Oxy_I	0.5	*Oxynoemacheilus leontinae*	ADM1019
Poeciliidae	*Gambusia affinis* ^†^	14	Ga.af_A	0.79	*Gambusia holbrooki*	AAC2757
			Ga.af_B	0.21	*"*	"
	*Poecelia* sp.^†^	3	Po.ve_A	0.33	*Poecilia velifera/latipinna/sphenops*	AAD1756
			Ga.af_A	0.33	*Gambusia holbrooki*	AAC2757
			Ga.af_B	0.33	*"*	"
	*Poecilia velifera* ^†^	2	Po.ve_A	1	*Poecilia velifera/latipinna/sphenops*	AAD1756
	*Xiphophorus* sp.^†^	2	Xi.sp._A	1	*Xiphophorus hellerii*	AAB8020

All collected barcode sequences and their respective sample data were deposited in the BOLD and GenBank databases (Table S1 and project FWISR in BOLD). The 205 sequences included 71 different barcodes (i.e., unique haplotypes), out of these, 26 barcodes belonging to 13 taxonomic groups were new additions to the BOLD database (Table 1). For the two native species, *Pseudophoxinus kervillei* (Pellegrin, 1911) and *Oxynoemacheilus dori* (Goren and Bănărescu, 1982), all six DNA barcodes deposited were not previously recorded in the BOLD database (Table 1). For 29 of the 32 taxonomic groups, more than one specimen was barcoded and for 18 of them, more than one haplotype was found. The lowest haplotype frequency was 9%, suggesting a relatively high abundance even for rare haplotypes (Table 1).

The utility of using DNA barcodes for identification of freshwater fish species in Israel was tested by comparing the molecular identification of all 205 specimens obtained here to their morphological identification. First, DNA barcodes were queried against BOLD database yielding a likely DNA‐based species identification, including percent sequence similarity between query sequence and best BOLD hits. A perfect match between morphological and DNA barcoding identification was obtained for 116 (56.6%) specimens, however, for 89 specimens, the best match records in BOLD either added a new haplotype for the same species or indicated discrepancies with the morphological identification (Table 1). Secondly, COI sequences were clustered using the RESL algorithm to delineate putative OTUs based on DNA barcodes. The analysis yielded 30 OTUs compared to 32 morphologically identified taxonomic groups, indicating further discrepancies between morphology and DNA barcoding. Taken together, discrepancies were found for samples falling into five genera of native species (*Acanthobrama*, *Garra*, *Pseudophoxinus*, *Oxynoemacheilus* and *Aphanius*). To investigate these discrepancies, genetically similar DNA barcodes with their species identification and sampling location were downloaded from BOLD and analyzed together with our barcodes and sample information.

### 3.2 Revisiting the genus *Acanthobrama*

**FIGURE 2:**
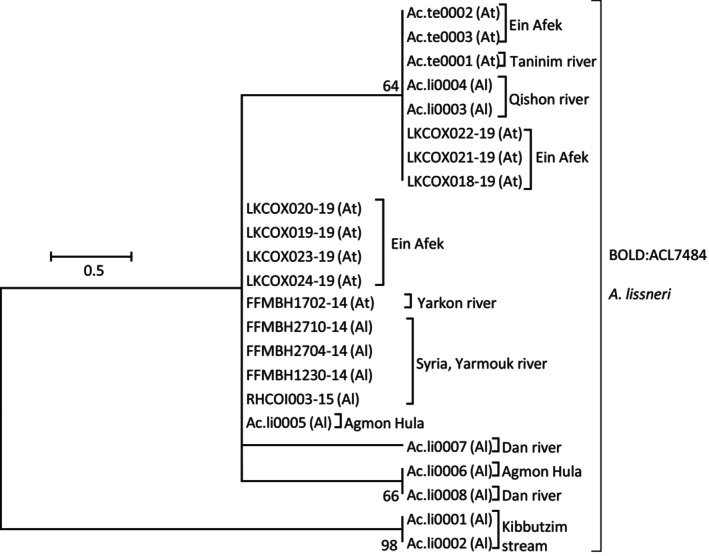
Haplotype phylogeny for *Acanthobrama* spp. samples. Numbers next to nodes are % bootstrap support. Samples of this study are named by study ID (Table S1) and BOLD samples by their Process ID. Species (as identified morphologically for study samples or as deposited by user for BOLD samples) in parentheses: At—*A. telavivensis* and Al—*A. lissneri*. Sampling sites in Israel are listed next to sample names, if the sampling site is outside of Israel sampling country is also mentioned. Suggested taxonomic cluster based on RESL algorithm for sequence‐based species delineation is listed next to sample names, by the BOLD BIN accession number.

For *Acanthobrama* species, 11 and 12 sequences, for *Acanthobrama telavivensis* Goren, Fishelson and Trewavas, 1973, and *Acanthobrama lissneri* Tortonese, 1952, respectively, were aligned and analyzed (File S2). All DNA barcodes of both species clustered to a single OTU, corresponding to BIN accession “BOLD:ACL7484.” In concordance, the NJ tree grouped together samples of *A. lissneri* from the Eastern watershed habitats in Israel and Syria (Yarmouk River) with samples of *A. telavivensis* from the Western watershed habitats in Israel. The tree suggested separation of only two samples from Kibbutzim stream in Beit She'an valley (Eastern watershed), identified as *A. lissneri* (Figure 2).

The K2P distances within *A. telavivensis* were low (0–0.2%) and slightly higher within *A. lissneri* (0–1.2%). Excluding the Kibbutzim stream samples, which were identified as *A. lissneri*, reduced the maximal K2P distance within all other samples to 0.5%, while the K2P distance between Kibbutzim stream samples to all others was 0.9%–1.2%, supporting the divergence of the Kibbutzim stream population on one hand and on the other, the high genetic similarity between the two morphologically identified species.

### 3.3 Revisiting the genus *Garra*

**FIGURE 3:**
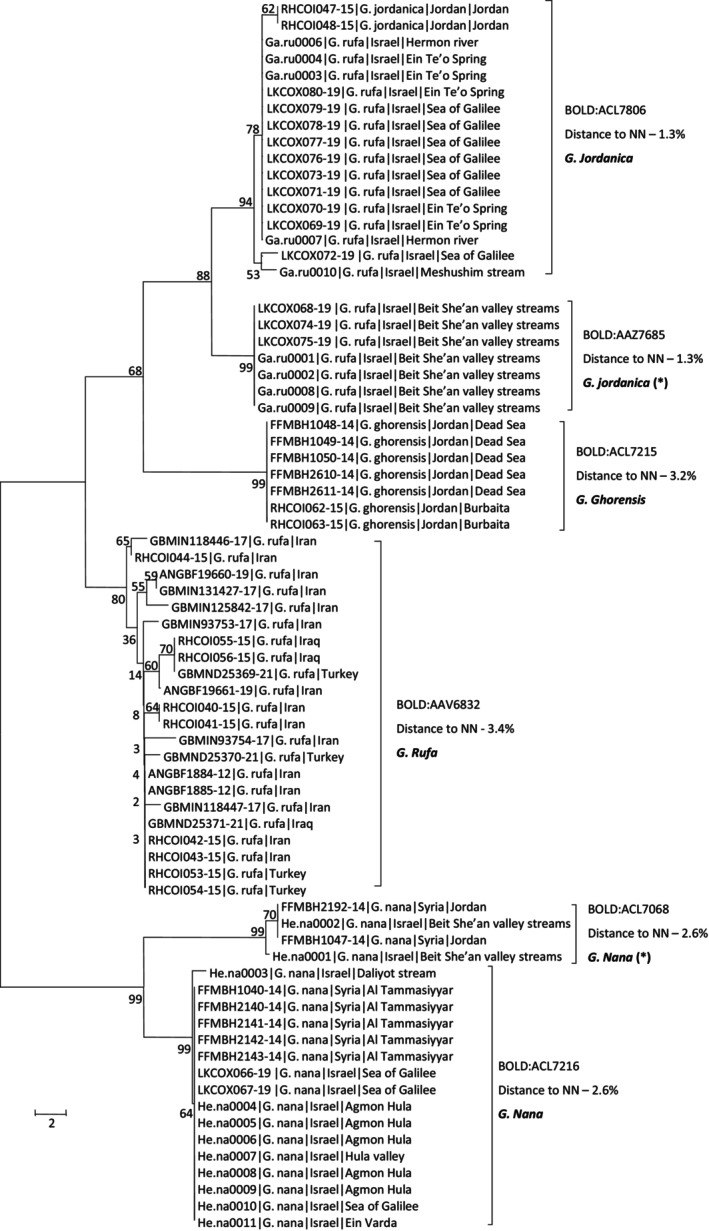
Haplotype phylogeny for *Garra* spp. samples. Numbers next to nodes are % bootstrap support. Samples of this study are named by study ID (Table S1) and BOLD samples by their Process ID. Following sample ID are species (as identified morphologically for study samples or as deposited by user for BOLD samples) and sampling country and site (for regional samples). Suggested taxonomic clusters based on RESL algorithm for sequence‐based species delineation are listed next to sample names, by their BOLD BIN accession numbers. The distance to the nearest neighbor (NN), and suggested taxonomy based on barcoding. Asterisks (*) next to suggested species names imply that we hypothesize that these specimens might need to be described as a new species.

For *Garra* species, 44, 20, seven and two sequences of *Garra rufa* (Heckel, 1843), *Garra nana* (Heckel, 1843), *Garra ghorensis* Krupp, 1982 and *Garra jordanica* (Hamidan et al., 2014), were analyzed (File S3). The four nominal *Garra* species clustered into six OTUs by the RESL algorithm in accordance with the NJ tree clustering (Figure 3). The first main cluster of the NJ analysis included specimens identified as *G. nana*, which further split into two OTUs. One (BIN accession BOLD:ACL7216) included samples from the Sea of Galilee itself and from sites northern to it (northern Jordan River basin, Israel and Al Tammasiyyar, Syria) and the other (BIN accession BOLD:ACL7068), included samples from south and east to the Sea of Galilee (Beit She'an valley streams, Israel and Jordan, Syria). The minimal genetic distance between these two OTUs was 2.6%.

The second major cluster of the tree split into four subclusters, which perfectly correspond to the remaining four suggested UTOs (Figure 3). One cluster included samples of *G. rufa* from the northern Jordan River basin, Israel and *G. jordanica* samples from Jordan (BIN accession BOLD:ACL7806). The second cluster included samples of *G. rufa* from Beit She'an valley streams (BIN accession BOLD:AAZ7685). The minimal genetic distance between these two OTUs was 1.3% and could be considered on the high range of intraspecific distances. A third cluster consisted of BOLD samples of *G. ghorensis* from the southern Dead Sea basin, Jordan (BIN accession BOLD:ACL7215). The minimal genetic distance to the nearest neighbor was 3.2%. A fourth, more separated, cluster included samples of *G. rufa* from other Asian countries (Turkey, Iraq and Iran; BIN accession BOLD:AAV6832) and their distance to the nearest neighbor was 3.4%.

### 3.4 Revisiting the genus *Pseudophoxinus*

**FIGURE 4:**
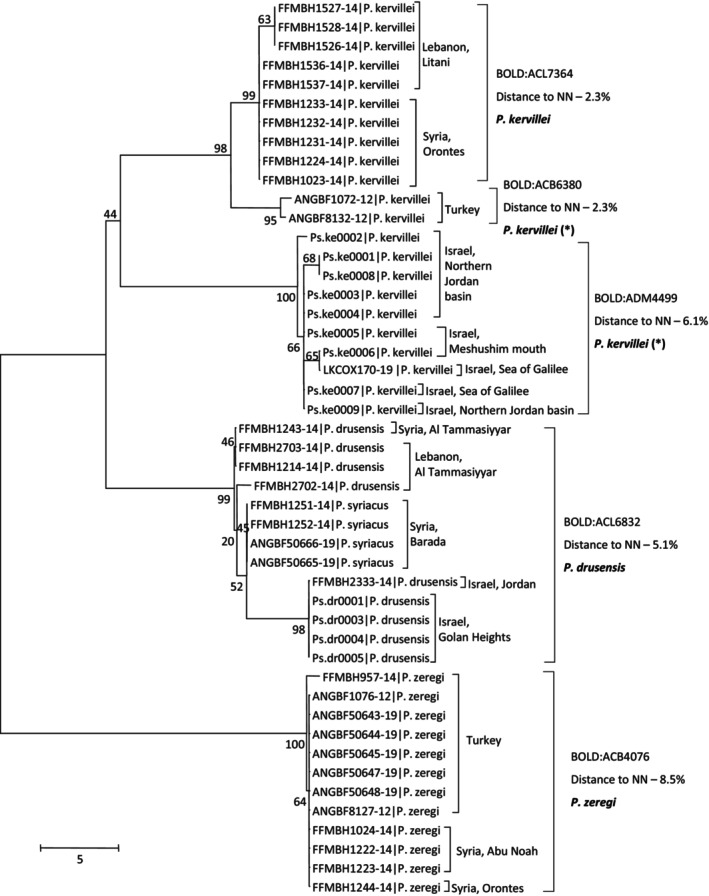
Haplotype phylogeny for *Pseudophoxinus* spp. samples. Numbers next to nodes are % bootstrap support. Samples of this study are named by study ID (Table S1) and BOLD samples by their Process ID. Following sample ID are species (as identified morphologically for study samples or as deposited by user for BOLD samples) and sampling country and region (for regional samples). Suggested taxonomic clusters based on RESL algorithm for sequence‐based species delineation are listed next to sample names, by their BOLD BIN accession numbers. The distance to the nearest neighbor (NN), and suggested taxonomy based on barcoding. Asterisks (*) next to suggested species names imply that we hypothesize that these specimens might need to be described as a new species.

For *Pseudophoxinus* species, 22, nine, four, and 12 sequences of *Pseudophoxinus kervillei* (Pellegrin, 1911), *Pseudophoxinus drusensis* (Pellegrin, 1933), *Pseudophoxinus syriacus* (Lortet, 1883), and *Pseudophoxinus zeregi* (Heckel, 1843), were aligned and analyzed (File S4). Accessions of these four nominal species clustered into five OTUs by the RESL algorithm and perfectly aligned with the NJ tree clustering (Figure 4). All samples of *P. zeregi* clustered together to a single OTU (BIN accession BOLD:ACB4076), and separately from all others in the NJ analysis (distance to nearest neighbor of 8.5%). The next split separated specimens morphologically identified as *P. drusensis* and *P. syriacus*, which also clustered to a single OTU (BIN accession BOLD:ACL6832), the distance of this OTU to nearest neighbor was 5.1%. Within this OTU, however, *P. syriacus* and *P. drusensis* samples from Syria and Lebanon were similar, while Israeli *P. drusensis* samples were slightly separated (Figure 4), with K2P distances in the range of 1.2%–1.5%. Interestingly, the remaining 22 samples, all morphologically identified as *P. kervillei*, were delineated into three OTUs, represented by separate clusters in the NJ tree (Figure 4). Two clusters were closer together (minimal genetic distance of 2.3%), one included samples from Lebanon and Syria (BIN accession BOLD:ACL7564) and the other samples from Turkey (BIN accession BOLD:ACB6380). A third cluster, considerably further away from other samples (distance to nearest neighbor of 6.1%), consisted of all samples from Israel (BIN accession BOLD:ADM4499).

### 3.5 Revisiting the genus *Oxynoemacheilus*

**FIGURE 5:**
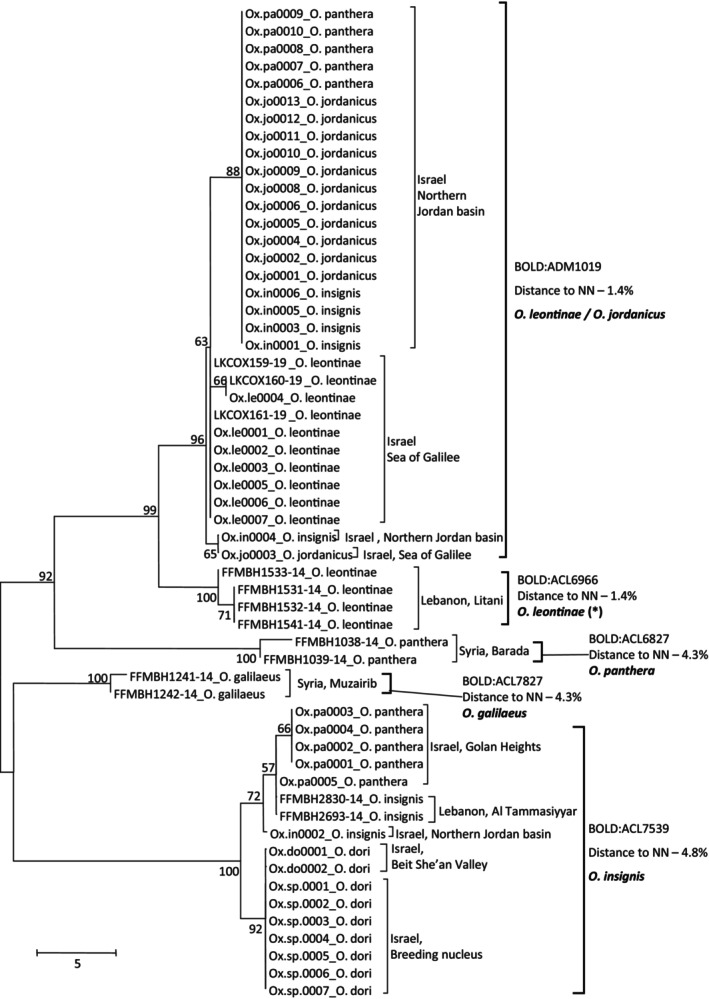
Haplotype phylogeny for *Oxynoemacheilus* spp. samples. Numbers next to nodes are % bootstrap support. Samples of this study are named by study ID (Table S1) and BOLD samples by their Process ID. Following sample ID are species (as identified morphologically for study samples or as deposited by user for BOLD samples) and sampling country and region. Suggested taxonomic clusters based on RESL algorithm for sequence‐based species delineation are listed next to sample names, by their BOLD BIN accession numbers. The distance to the nearest neighbor (NN), and suggested taxonomy based on barcoding. Asterisks (*) next to suggested species names imply that we hypothesize that these specimens might need to be described as a new species.

Samples of five morphologically identified *Oxynoemacheilus* species were collected from Israeli sites: *Oxynoemacheilus insignis* (Heckel, 1843), *Oxynoemacheilus panthera* (Heckel, 1843), *Oxynoemacheilus leontinae* (Lortet, 1883), *Oxynoemacheilus jordanicus* (Bănărescu & Nalbant, 1966), and *Oxynoemacheilus dori* (Goren and Bănărescu, 1982). For these species (respectively), eight, 12, 14, 12, and nine DNA barcodes (from this study and BOLD) were aligned and analyzed (File S5). BOLD sequences added another species, *Oxynoemacheilus galilaeus* (Günther, 1864), from Syria. Genetic distances were within range of intraspecific variation (up to 2%) for each of *O. leontinae*, *O. galilaeus*, *O. jordanicus* and *O. dori*. In contrast, high intraspecific variation was found within *O. panthera* and *O. insignis* (up to 8.6% and 8.1%, respectively). Furthermore, the six morphologically identified species clustered into five OTUs by the RESL algorithm, which corresponded to four main tree clusters in the NJ analysis (Figure 5).

One cluster contained a single OTU consisted of two *O. galilaeus* samples (BIN accession BOLD:ACL7827) with distance to nearest neighbor of 4.3%. Another cluster included *O. panthera* from the Golan Heights, Israel, *O. insignis* fish from Al Tammasiyyar, Lebanon, a single *O. insignis* specimen (Ox.in0002) from the Dan River (Northern Jordan River basin), Israel and *O. dori* from Ein Malkoach, Beit She'an valley, Israel (BIN accession BOLD:ACL7539), with a minimal distance to nearest neighbor of 4.8%. A third cluster contained two *O. panthera* samples from Barada, Syria (BIN accession BOLD:ACL6827), with distance to nearest neighbor of 4.3%.

Lastly, a fourth cluster included two OTUs, with distance to nearest neighbor of 1.4% to one another. One OTU with samples identified as *O. leontinae* from Litani River, Lebanon (BIN accession BOLD:ACL6966), and another with samples morphologically identified to four species: *O. panthera*, *O. jordanicus*, and *O. insignis* from the northern Jordan River basin and *O. leontinae* from the Sea of Galilee, Israel (BIN accession BOLD:ADM1019). Thus, *O. panthera* and *O. insignis*, which had high intraspecific variation, were each included in three and two OTUs, respectively. In contrast, samples of *O. dori* from Israel were significantly separated by the NJ tree, but were within an OTU with *O. panthera* and *O. insignis* fish, with a distance to nearest neighbor of only 0.7%.

### 3.6 Revisiting populations of *Aphanius mento*

**FIGURE 6:**
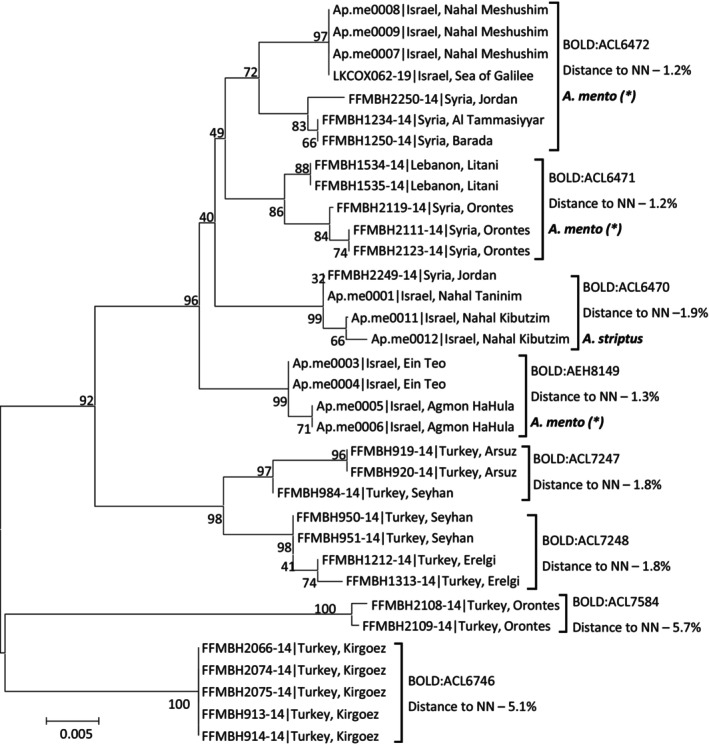
Haplotype phylogeny for *Aphanius mento* samples. Numbers next to nodes are % bootstrap support. Samples of this study are named by study ID (Table S1) and BOLD samples by their Process ID. Following sample ID is sampling country and site. Suggested taxonomic clusters based on RESL algorithm for sequence‐based species delineation are listed next to sample names, by their BOLD BIN accession numbers. The distance to the nearest neighbor (NN), and suggested taxonomy based on barcoding. Asterisks (*) next to suggested species names imply that we hypothesize that these specimens might need to be described as a new species.

For *Aphanius mento* (Heckel, 1843), DNA barcodes of 11 samples from Israel in addition to 23 BOLD records from Syria, Lebanon and Turkey were aligned and analyzed (File S6). Even though all samples were morphologically identified as *A. mento*, eight different OTUs were suggested by the RESL algorithm and by the NJ tree (Figure 6). The tree separated between fish from Turkey and fish from closer countries (Syria, Lebanon and Israel, Figure 6). The fish from Turkey had a K2P distances range of 3.5%–7.2% relative to fish outside Turkey. The samples from Turkey were further divided into four cluster on the NJ tree, representing four suggested OTUs (Bin accessions BOLD:ACL7248, ACL7247, ACL7584, ACL6746, Figure 6), and possibly, four separate species. Given the divergence of fish in Turkey, we further focused on the specimens from Israel and its neighboring countries excluding Turkey.

Samples outside Turkey clustered into four OTUs with a distinctive geographic separation (Figure 6). The first OTU (BIN accession BOLD:ACL6472) included samples from the Sea of Galilee and mouth of Meshushim stream, Israel and samples from the Syrian part of the northern Jordan River basin. Within this OTU, samples from Israel shared a single barcode that was 1.1%–1.6% different from the samples from Syria. The second OTU (BIN accession BOLD:ACL6471) included samples from Orontes River, Syria and Litani River, Lebanon. A third, more separate OTU (BIN accession BOLD:ACL6470), included samples from Beit She'an valley (Kibbutzim stream) in the east of Israel, Mediterranean Sea coastal streams (Taninim river) in the west of Israel, and Syrian side of the southern Jordan River basin. The fourth OTU (BIN accession BOLD:AEH8149), had the strongest bootstrap support for separation (excluding Turkey) and included samples from northern Jordan River basin (Ein Teo and Hula valley), Israel. The third OTU had a distance to nearest neighbor of 1.9% compared to 1.2–1.3% between the other three (excluding Turkey).

## 4 DISCUSSION

### 4.1 Utility of the COI DNA barcoding database

In this study, DNA barcodes were produced for 21 native and seven non‐native species, and together with barcodes previously reported from the region (Geiger et al., 2014; Shirak et al., 2009; Tadmor‐Levi, Borovski, et al., 2022), a comprehensive DNA barcode reference library for the freshwater fish of Israel and the surrounding region was created, which is now publicly available in BOLD. This resource serves as a reference baseline in supporting future taxonomic and ecological research as well as conservational measures. From a practical perspective, confidently identified barcode sequences serve as references, permitting identification of fish specimens, fish products or DNA in environmental samples to species. The availability of these DNA barcodes has already become valuable for studies on the ecology of some species (Borovski et al., 2018; Tadmor‐Levi, Borovski, et al., 2022; Tadmor‐Levi, Cummings, et al., 2022) and can be further used to support species monitoring and conservation policy decisions. The value of these data extends to studies on this species‐rich region, since the distribution of most barcoded species goes beyond Israel, as demonstrated here for several species. Since interspecific is larger than intraspecific variation, new haplotypes that could still be discovered are likely to be accurately identified to their correct species when queried against this database.

### 4.2 Correspondence between morphological and DNA‐based species identification

All specimens were identified based on morphology. For 116 samples out of 205, our DNA barcoding analysis confirmed the morphological species identification. Molecular identification relies on previous database records and thus, is more established for widely distributed species. Indeed, we found such matches in our data for species distributed also outside the region such as *Cyprinus carpio* Linnaeus 1758, *Mugil cephalus* Linnaeus 1758, *Chelon ramada* (Risso, 1827), *Morone chrysops* (Rafinesque, 1820), *Amatitlania nigrofasciata* (Günther, 1867), *Clarias gariepinus* (Burchell, 1822) and *Anguilla anguilla* (Linnaeus, 1758). Additionally, for 13 native species, matching database records from Israel existed from earlier studies (Geiger et al., 2014; Shirak et al., 2009; Tadmor‐Levi, Borovski, et al., 2022). Nevertheless, for 89 samples, the best match records on BOLD either added information or indicated discrepancies with the morphological identification. Of those, 13 samples were identified morphologically only to their genus or family level and DNA barcoding complemented their identification to the specific level (Table 1).

Some discrepancies were for introduced species. For 14 samples, morphologically identified as *Gambusia affinis* (Baird and Girard, 1853), sampled from eight separate freshwater habitats, identification by BOLD records was as *Gambusia holbrooki* Girard, 1859. Both these species of mosquitofish were globally introduced to control mosquito swarms and often turned invasive (Ben‐Tuvia, 1981; Goren & Ortal, 1999). It is possible that into Israel, *G. holbrooki* was introduced rather than *G. affinis*. Two *Gambusia* samples were mistakenly identified as *Poecilia* sp., additional two samples that were morphologically identified as *Poecilia velifera* (Regan, 1914), were inconclusively identified by BOLD as either *P. velifera* or *Poecilia latipinna* (Lesueur, 1821) or *Poecilia sphenops* Valenciennes, 1846. These *Poecilia* alternatives belong all to non‐native fish that probably escaped from captivity into a stream pool in Beit She'an valley.

More importantly, however, disagreement between morphological and molecular identification were found for several native species belonging to five genera, *Acanthobrama*, *Garra*, *Pseudophoxinus*, *Oxynoemacheilus* and *Aphanius*. Integrating geographic and DNA barcoding information, for samples of this study and BOLD systems, allowed reconsidering their morphological species identification and consequently, the species list and distribution in Israel and the region. Reconsiderations made here are based mainly on DNA barcoding, and therefore, further investigations including more samples, thorough morphological examination and perhaps more genetic makers are called for to make formal taxonomic resolutions.

### 4.3 *Acanthobrama* species in Israel

Three native species of bleak were reported from Israel. *Acanthobrama lissneri*, inhabiting mainly the Jordan River system (Eastern watershed of Israel), *A. telavivensis*, inhabiting some Mediterranean coastal rivers (Western watershed of Israel) and *Mirogrex terraesanctae* (Steinitz, 1952) (formerly *Acanthobrama* terraesanctae), endemic to the Sea of Galilee, which was already analyzed in a different study (Tadmor‐Levi, Borovski, et al., 2022). Despite the geographic separation and morphological differences (Goren, 1973), DNA barcodes of all *A. lissneri* and *A. telavivensis* specimens formed a single unresolved cluster in the NJ analysis (Figure 2). The sequence from Kibbutzim stream in Beit She'an valley was the most divergent, with K2P distances to *A. lissneri* and *A. telavivensis* from other localities of 0.9%–1.2%. However, this is within the range empirically considered to be intraspecific variation (Ward, 2009), and this population clustered into the same OTU (BIN accession BOLD:ACL7484) as all other *Acanthobrama* samples. Thus, based on our DNA barcoding analyses, we suggest reconsidering the separate species status of *A. telavivensis*.

### 4.4 *Garra* species in Israel and the region

Currently, two species of *Garra* are considered to occur in Israel, *G. rufa* and *G. nana*, both found in the Jordan River basin and the Qishon River (coastal streams). Another species, *G. ghorensis*, was described from the basin south of the Dead Sea (Jordan and Israel) and is today considered to be extinct from Israel's side (Freyhof, 2014a; Hamidan & Mir, 2003). A more recent study, focused on *Garra* species in Jordan, described *G. jordanica*, a species inhabiting the Eastern (Jordanian) side of the southern Jordan River valley, north to the Dead Sea (Hamidan et al., 2014). Our DNA barcoding analyses have cast doubt on the validity of some of the species of *Garra* occurring in the region (Figure 3). First, in accordance with previous studies (Hamidan et al., 2014; Tadmor‐Levi, Borovski, et al., 2022), *G. rufa* from the northern Jordan River basin and Sea of Galilee Israel clustered together with the *G. jordanica* from Jordan and separate from *G. rufa* from other Asian countries (Iran, Iraq and Turkey, Figure 3). Thus, *G. rufa* from Israel have been misidentified and are *G. jordanica*. Furthermore, *G. jordanica* are divided into two separate BINs corresponding to two separate clusters in the NJ analysis, separating northern Jordan river basin from Beit She'an streams populations. Barcoding‐based species delineation suggests that these separate populations might be two different species, however distance to nearest neighbor between the two is only 1.3%. Thus, this separation is relatively weak, and additional data is needed to decide if these genetic differences justify splitting into two species.

Second, *Garra nana* populations in the Beit She'an valley and southern Jordan basin, Syria, formed completely separated OTUs and two separate clusters in the NJ analysis from the populations in the northern Jordan River basin from Israel and Syria (Figure 3), with considerable genetic distance between nearest neighbors of 2.6%. *Garra nana* was first described from Damascus, Syria and thus, based on geographic distribution and our DNA barcoding analyses we hypothesize that fish from the Northern Jordan basin in Israel (and Syria), are the originally described *G. nana*, while more southern fish from the streams of the Beit She'an Valley could be considered a separate, potentially undescribed, species.

### 4.5 *Pseudophoxinus* species in Israel and the region

*Pseudophoxinus zeregi* samples from Turkey and Syria were genetically distinct (Figure 4); hence, *P. zeregi* is likely a separate species from the other three. *Pseudophoxinus drusensis* samples from Israel were grouped in one OTU with *P. drusensis* samples from Syria and Lebanon and *P. syriacus* samples from Syria (Figure 4). Thus, *P. drusensis* is a separate regional species with isolated and slightly genetically different (separated clusters in the NJ analysis) populations in Israel compared with Syria and Lebanon. *Pseudophoxinus syriacus* was reported as a small endemic population in Syria that is considered critically endangered or even extinct by the IUCN red list (Freyhof, 2014d). Based on our barcoding analyses, *P. syriacus* might not necessarily be a separate species from *P. drusensis*, and therefore, its current conservation status might change accordingly.

*Pseudophoxinus kervillei* from Israel had five different barcode haplotypes forming a single OTU and clustering into a single BIN, with a nearest‐neighbor distance of 6.1% to the *P. kervillei* samples from outside of Israel (Figure 4). *Pseudophoxinus kervillei* samples from outside Israel were also split into two clusters, one including samples from Turkey and another samples from Syria and Lebanon. These two clusters also correspond to two separate BINs on BOLD, the closest genetic distance between them was 2.3%. *Pseudophoxinus kervillei* was originally described from around Homs, Syria; therefore, the cluster containing *P. kervillei* specimens from Syria and Lebanon probably corresponds to the original *P. kervillei*. Based on DNA barcoding and geographic distribution, “*P. kervillei*” in Turkey are possibly of a separate species, and “*P. kervillei*” in Israel are of another different species, yet to be described.

### 4.6 *Oxynoemacheilus* species in Israel and the region

The most complex inconsistencies between current morphological identifications and DNA barcoding species identifications were found in the *Oxynoemacheilus* spp. group (Figure 5). Currently, five species are known to occur in Israel: *O. insignis*, *O. panthera*, *O. leontinae*, *O. jordanicus*, and *O. dori*. Another species, *O. galilaeus* is a rare endemic species, reported extinct from Israel and considered critically endangered in Lake Muzairib, Syria, especially given the recent water pollution and reduced water levels of this Lake (Freyhof, 2014b). *Oxynoemacheilus galilaeus*, represented here by two samples from Lake Muzairib, Syria, was the only species with full concordance between morphological identifications and DNA barcoding analyses. Samples clustered together (BIN accession BOLD:ACL7827) with a considerable distance to nearest neighbor of 4.3%, supporting a separate species status. All other species deserve further reconsiderations.

*Oxynoemacheilus panthera* is a species described from Damascus in Syria, with a wider reported distribution in Lebanon, Israel, Syria, Turkey, and Iraq, yet it is considered an endangered species (Freyhof, 2014c). In our analyses, samples of *O. panthera* were divided into three separate clusters in the NJ analysis and three BOLD BINs. One cluster and BIN (accession BOLD:ACL6827) contained specimens from Barada river in Syria and was separated from all other samples (distance to nearest neighbor of 4.3%). Therefore, based on our DNA barcoding analyses, we hypothesize that only the population from Barada, Syria, should be identified as *O. panthera*, while the populations in Israel, one from the Golan heights and another from the Northern Jordan River basin represent two different species. It seems like both *O. galilaeus* and *O. panthera* are endangered species with limited distributions outside Israel. The former went extinct in Israel, and the latter is probably a misidentification in Israel.

*Oxynoemacheilus jordanicus* and *O. dori* are both endemic to Israel and were identified as separate species from *O. insignis* relatively recently (Banarescu et al., 1982; Banarescu & Nalbant, 1966; Krupp & Schneider, 1989). On BOLD systems, their COI barcodes were identified as *O. insignis* and they are referred to as such, or as subspecies of *O. insignis*, also in other databases such as FishBase, ITIS, IUCN, GBIF and NCBI. In our data, *O. jordanicus* specimens that were sampled from the northern Jordan River basin are combined into one BIN with *O. insignis*, *O. panthera*, and *O.leontinae* samples from the same location (accession BOLD:ADM1019) and therefore, likely do not justify a separate species status. *Oxynoemacheilus dori* has a natural small population in Ein Malkoach stream in the Beit She'an Valley. Due to extinction concerns, a breeding nucleus was established in Israel to support the natural population. The DNA barcodes of all samples of *O. dori* were identical, forming a subcluster in the NJ tree. However, these samples, together with samples identified as *O. panthera* from the Golan Heights and *O. insignis* from the northern Jordan River basin in Israel and Lebanon, all cluster into a single OTU (BIN accession BOLD:ACL7539). Therefore, the separate species status of *O. dori* is not supported by our barcoding analyses and we hypothesize that all samples within BIN accession BOLD:ACL7539 are of a single species, possibly *O. insignis*, distributed in Beit She'an valley, and Golan Heights, Israel and further east in Al Tammasiyyar, Lebanon.

Another species, *O. leontinae*, is endemic to the Jordan and Litany River systems and morphologically distinct from the other species (Krupp & Schneider, 1989). *Oxynoemacheilus leontinae* specimens from the Sea of Galilee, Israel clustered together in one BIN with *O. jordanicus*, *O. insignis*, and *O. panthera* from the northern Jordan River Basin, Israel. Interestingly, other samples identified as *O. leontinae* from the Litani River, Lebanon, are the sister group but in a separate BOLD BIN with a distance to nearest neighbor of 1.4%. Since *O. leontinae* was originally described from the Sea of Galilee, DNA barcoding suggests that fish currently identified as *O. leontinae* from Litani River, Lebanon, might be of a different species.

Taken together, of the six reported species, our DNA barcoding analyses suggested that only five *Oxynoemacheilus* species (OTUs) exist in the region, two of them in Israel. Interestingly, Krupp & Schneider (1989) also suggested that only two species exist in the region, *O. insignis* and *O. leontinae*, which differ by significant distinctive morphologic characteristics. However, dividing the specimens from Israel based on the published key by Krupp & Schneider (1989), only separates specimens defined as *O. leontinae* from all others and does not correlate with the division suggested by the barcoding analyses (Figure 5). Therefore, combining the two methods (barcoding and morphology), may suggest that three species exist in Israel: *O. leontinae* in the Sea of Galilee, *O. jordanicus* in the Northern Jordan River basin and *O. insignis* in the Golan Heights and the Beit She'an Valley. From the available barcode records, these species are found also in Lebanon, whereas *O. panthera* exists only in Syria and possibly also *O. galilaeus*, which went extinct in Israel.

Three samples, morphologically identified as *O. insignis*, one (Ox.in0002) from the Northern Jordan River basin, Israel, and two from Al Tammasiyyar, Lebanon, are part of the OTU that we suggested to identify as *O. insignis*, which includes more samples from eastern Golan Heights and southern Beit She'an Valley, Israel. It might indicate that *O. insignis* and *O. leontinae* suggested in Israel are partly overlapping in their distribution range at the northern Jordan River basin in Israel. However, more focused research with further and deeper sampling in all locations, thorough morphological examinations, and further DNA analyses (possibly with additional markers) of *Oxynoemaceilus* populations in Israel is called for describing these species in Israel correctly.

### 4.7 Geographic and genetic gradients of *Aphanius mento* populations in Israel and the region

Populations of *A. mento* have been reported from Iraq, Jordan, Syria, Israel, Lebanon, and Turkey. Our DNA barcoding analyses suggested that the fish in Turkey are not of the same species as the rest and probably deserve species recognitions of their own. The populations from the nearby region exhibited genetic divergence by geographic regions, which could support consideration of up to four species (Figure 6). Until now, based on geographic distribution and morphology, two sub‐species were reported from Israel: *Aphanius mento mento* in the Sea of Galilee and northern Jordan River basin, and *A. mento striptus* in southern Jordan River basin (including Beit She'an valley streams) and Mediterranean coastal streams (Goren, 1974). In concordance with *A. mento striptus*, the most genetically distant OTU (distance to nearest neighbor of 1.9%) included samples from the Beit She'an valley (Kibbutzim stream) in the east of Israel and Mediterranean Sea coastal streams (Taninim River) in the west of Israel and from the Syrian side of the southern Jordan River basin (BIN accession BOLD:ACL6470). Thus, based on our DNA barcoding analyses, geographic distribution and morphological characteristics (Goren, 1974), we suggest that this population can be considered as *Aphanius striptus* instead of a subspecies. The rest of the regional samples cluster into three OTUs by the DNA barcoding‐based species delineation procedure, although they have lower genetic distances to other clusters. Additionally, samples from the Sea of Galilee and bordering streams east of the lake clustered into one OTU (BIN accession BOLD:ACL6472), while specimens from streams north of the lake that are also drained to the Sea of Galilee formed a different cluster (BIN accession BOLD:AEH8149), and thus, there is a potential for an overlap in distribution. A larger sampling size from the Sea of Galilee might reveal if the two populations exist in the Sea of Galilee, or even hybridize with each other. In any case, the genetic divergence gradient found here requires larger sample sizes and possibly additional genetic markers to more accurately resolve the species and populations of *A. mento* in this region.

### 4.8 Uniqueness of populations in Israel and biodiversity “hot spots”

It is clearly evident from this study that the advantages of DNA barcoding go beyond just adding another layer of genetic information for consideration of taxonomy. Genetic variation continuously accumulates between populations and species as a function of time since reproductive separation of OTUs. Therefore, it provides clues also on intraspecific variation and unique populations. Although a better view of genetic variation can be obtained by using other DNA‐based methods, DNA barcoding can still provide genetic information valuable for decisions on conservation of species and populations. In this study, intraspecific COI variation was found for 18 of 32 taxonomic groups, among which were 26 new barcodes for 13 previously barcoded species, indicating that fish populations in Israel contain unique genetic variation. This genetic uniqueness is obvious also from the NJ trees and species delineation analyses done for groups with discrepancies between morphological and molecular identification. Freshwater habitats in Israel are unique and isolated, and many of the fish populations are relatively small and on the edge of the species geographic distribution, all are factors that can contribute to divergence.

In addition, genetic variation patterns highlighted biodiversity “hot spots,” as target regions for implementing conservation measures. Clearly, the Sea of Galilee, being the largest natural surface freshwater body in the Middle East, is also a key habitat supporting the largest regional populations of about 18 native species, of which three are endemic to the lake, and eight significant non‐native species (Goren & Ortal, 1999; Ostrovsky et al., 2014; Tadmor‐Levi, Borovski, et al., 2022). DNA barcoding studies, including this one, highlighted the unique genetic variation found in fish populations of the lake (Borovski et al., 2018; Shirak et al., 2009; Tadmor‐Levi, Borovski, et al., 2022; Tadmor‐Levi, Cummings, et al., 2022). Our analyses of DNA barcode sequence variation, presented evidence that may undermine the species status of some native lake populations including *G. nana*, *G. rufa*, *A. lisnneri*, *P. kervillei*, *O. leontinae*, and *A. mento*. A previous study highlighted also the unique genetic variation of the *Sarotherodon galilaeus* (Linnaeus, 1758) population in the lake (Borovski et al., 2018) and here, we identified new DNA barcode haplotypes for several resident species like *Oreochromis aureus* (Steindachner, 1864), *Coptodon zillii* (Gervais, 1848), and *Capoeta damascina* (Valenciennes, 1842).

This study made it clear that the Beit She'an valley region is also such a biodiversity “hot spot.” Although close to the Jordan River and Sea of Galilee, it is an isolated ecosystem (Golani et al., 2023; Stein, 2014). Despite its geographical proximity, the genetic divergence of the populations in Beit She'an valley from other eastern samples is larger than that between shared eastern and western populations, which are more geographically distant. A list of about 14 native fish species, very similar to that in the Sea of Galilee, was reported in this region (Goren & Ortal, 1999), but for five of these species unique local haplotypes were identified. Based on our DNA barcoding analyses, we suggest to re‐assess the species status for resident native populations of *G. nana*, *O. dori*, *A. mento*, *A. lissneri*, and *G. rufa*.

Taken together, DNA barcoding added a layer of continuous genetic divergence information, which when integrated with morphological species identification and patterns of geographic distribution, enabled revisiting the species list of freshwater fish in Israel and the nearby region, suggested reconsiderations in regional species and highlighted the Sea of Galilee and Beit She'an valley streams as species‐rich habitats supporting populations with distinct genetic variation, hence as targets for biodiversity monitoring and protection.

## AUTHOR CONTRIBUTIONS

**Roni Tadmor‐Levi:** Data curation (lead); formal analysis (lead); investigation (equal); methodology (lead); validation (lead); visualization (lead); writing – original draft (equal); writing – review and editing (equal). **Tamar Feldstein‐Farkash:** Conceptualization (supporting); data curation (supporting); investigation (supporting); methodology (supporting); resources (equal); writing – original draft (supporting); writing – review and editing (supporting). **Dana Milstein:** Conceptualization (equal); data curation (supporting); project administration (lead); resources (equal); writing – review and editing (supporting). **Daniel Golani:** Data curation (supporting); investigation (supporting); methodology (supporting); resources (supporting); validation (supporting); writing – review and editing (supporting). **Noam Leader:** Conceptualization (equal); funding acquisition (lead); project administration (supporting); resources (equal); writing – review and editing (supporting). **Menachem Goren:** Conceptualization (equal); data curation (equal); formal analysis (supporting); methodology (equal); resources (equal); writing – review and editing (supporting). **Lior David:** Conceptualization (equal); formal analysis (equal); funding acquisition (equal); investigation (equal); methodology (equal); resources (equal); supervision (lead); writing – original draft (equal); writing – review and editing (equal).

## Supporting information


File S1

File S2

File S3

File S4

File S5

File S6

Data S1

## Data Availability

The COI DNA sequences generated here and their relevant sample data were deposited under the project name FWISR on BOLD (https://www.boldsystems.org/, accessions FWISR001‐FWISR224) and to GenBank (https://www.ncbi.nlm.nih.gov/genbank/, accessions QQ991944–QQ992149) for the purpose of making the data widely available to the scientific community. Accession numbers are given in Table S1 and alignment files for each taxonomic group in disagreement are given in [Link], [Link], [Link], [Link], [Link] supplementary files. Benefits from this research accrue from the sharing of our data as described above.

## APPENDIX 1

### 1.1

See Table A1.

**TABLE A1:** Native freshwater fish species of Israel (modified from Goren & Ortal, 1999) and their current status.

Family	Species	Origin	Habitat (in Israel)	Comments
Anguilidae	*Anguilla anguilla*	Mediterranean	Coastal system	Unintentionally introduced in the Sea of Galilee
Cyprinidae	*Acanthobrama lissneri*	Middle east	Jordan Valley, Qishon River	Highly similar to *Acanthobrama telavivensis*
	*Acanthobrama telavivensis*	Middle east	Coastal system excluding Qishon River	Highly similar to *Acanthobrama lissneri*, currently exsists only in Yarkon River and Ein Afek springs
	*Carasobarbus canis*	Asia‐Africa	Jordan Valley	
	*Luciobarbus longiceps*	Asia	Jordan Valley	Was not sampled in this study as was previously covered in a study by Tadmor‐Levi, Borovski, et al. (2022)
	*Capoeta damascina*	Middle east	Coastal system and Jordan Valley	
	*Garra ghorensis*	Asia	Southern dead sea basin	Considered extinct from Israel
	*Garra rufa*	Asia	Coastal and Jordan systems	Should be *Garra jordanica*
	*Garra nana*	Middle east	Jordan Valley, Qishon River	Consider splitting into two separate species
	*Mirogrex hulensis*	Middle east	Hula Valley	Considered extinct
	*Mirogrex terrasanctae*	Middle east	Sea of Galilee	Was not sampled in this study as was previously covered in a study by Tadmor‐Levi, Borovski, et al. (2022)
	*Pseudophoxinus drusensis*	Asia‐Europe	Golan Heights	Highly similar to *Pseudophoxinus syriacus*
	*Pseudophoxinus kervillei*	Asia‐Europe	Jordan Valley	Population in Israel is probably a different species
Nemacheilidae	*Oxynoemacheilus dori*	Asia‐Europe	Beit She'an Valley	Suggested to be a junior synonym for *Oxynoemacheilus insignis*
	*Oxynoemacheilus jordanicus*	Asia‐Europe	Jordan Valley	
	*Oxynoemacheilus panthera*	Asia‐Europe	Golan Heights	Probably mis‐identified in Israel
	*Oxynoemacheilus leontinae*	Asia‐Europe	Sea of Galilee	Similar DNA barcodes to *Oxynoemacheilus jordanicus*, however with distinct morphological differences
	*Oxynoemacheilus insignis*	Asia‐Europe	Jordan Valley	Some samples might be mis‐identified, further sampling is needed
	*Oxynoemacheilus galilaeus*	Asia‐Europe	Sea of Galilee, Hula Valley	Considered extinct from Israel
Claridae	*Clarias gariepinus*	Africa	Coastal and Jordan systems	
Cyprinodontidae	*Aphanius mento*	Asia	Coastal and Jordan systems	
	*Aphaniops richardsoni*	Red Sea	Dead Sea	Endemic to the springs around the dead sea, habitat was not covered during sampling
Mugilidae	*Mugil cephalus*	Mediterranean	Coastal system	Introduced in the Sea of Galilee
	*Chelon ramada*	Mediterranean	Coastal system	Introduced in the Sea of Galilee
Blenniidae	*Salaria fluviatilis*	Mediterranean	Sea of Galilee and central Coastal system	
Cichlidae	*Astatotilapia flavijosephi*	Africa	Sea of Galilee and Beit She'an Valley	
	*Oreochromis aureus*	Africa	Coastal system and Jordan Valley	
	*Oreochromis niloticus*	Africa	Southern coastal system	Natural populations are considered extinct from Israel
	*Sarotherodon galilaeus*	Africa	Coastal system and Jordan Valley	
	*Coptodon zillii*	Africa	Coastal system and Jordan Valley	
	*Tristramella sacra*	Africa	Sea of Galilee	Considered extinct
	*Tristramella simonis*	Africa	Sea of Galilee	Endemic to the Sea of Galilee, was not sampled in this study as was previously covered in a study by Tadmor‐Levi, Borovski, et al. (2022)
