# Supplementary material for: Revisiting the species list of freshwater fish in Israel based on DNA barcoding
Source: Ecol Evol. 2023 Dec 20;13(12):e10812. doi: 10.1002/ece3.10812 (PMC10731390; doi:10.1002/ece3.10812)
Supplement: Supplementary file 7 — Data S1 [file ECE3-13-e10812-s004.docx]

**Supplementary materials**

**Supplementary file 1 – Table S1** – List of 205 DNA barcoded samples, with details of study code, museum code, BOLD process ID, GenBank accessions ,morphological and molecular species identification, sampling site details and COI haplotype.

**Supplementary file 2 – *Acanthobrama* spp. Alignment fasta file.**

**Supplementary file 3 – *Garra* spp. Alignment fasta file.**

**Supplementary file 4 – *Pseudophoxinus* spp. Alignment fasta file.**

**Supplementary file 5 – *Oxynoemacheilus* spp. Alignment fasta file.**

**Supplementary file 6 – *Aphanius mento* Alignment fasta file.**
